# Moving Microbiome Science from the Bench to the Bedside: a Physician-Scientist Perspective

**DOI:** 10.1128/mSystems.00160-19

**Published:** 2019-05-28

**Authors:** Neeraj K. Surana

**Affiliations:** a Department of Pediatrics, Duke University, Durham, North Carolina, USA; b Department of Molecular Genetics and Microbiology, Duke University, Durham, North Carolina, USA; c Department of Immunology, Duke University, Durham, North Carolina, USA

**Keywords:** clinical therapeutics, microbiome, translation

## Abstract

The recognition over the past decade that nearly all diseases are associated with changes in the microbiome has raised hope that microbiome-based therapeutics may cure many human ailments. Billions of dollars are being poured into microbiome-oriented biotech companies, and the coming years will undoubtedly witness the approval of the first generation of these products. However, significant hurdles remain in expanding the pipeline and advancing these first-generation therapies. In this perspective, I will discuss the challenges related to identifying causal microbes, determining their mechanism of action, and characterizing the specific bacterial molecules required for disease protection. We are approaching these issues through a combination of clinical sampling, animal models, classic microbiology methodologies, and systems-based approaches. The field of microbiome research is on the cusp of being able to identify clinically actionable host-microbe relationships; increasing attention on identifying causal microbes and their bioactive factors will usher in the next generation of microbiome-based therapies.

## Abstract (precis)

The recognition over the past decade that nearly all diseases are associated with changes in the microbiome has raised hope that microbiome-based therapeutics may cure many human ailments. Billions of dollars are being poured into microbiome-oriented biotech companies, and the coming years will undoubtedly witness the approval of the first generation of these products.

## PERSPECTIVE

The numerous microbiome-disease associations identified thus far have generated a great deal of hope that understanding relevant host-microbe interactions will open the door to unlimited therapeutic applications (1). Microbiome-based therapies offer several potential benefits. Patients often view such treatment as more “natural” than conventional drug therapy and are therefore more likely to comply with it. Biologically, microbiome-based therapies are more likely to address one of the root causes of disease (microbial dysbiosis) rather than simply affecting the downstream sequelae. Finally, a given microbiome-based therapy may serve as a “polypill” that is effective against several different diseases stemming from similar microbial changes. Despite tremendous interest in therapeutically exploiting the microbiome, there have thus far been few clinical successes along these lines.

The most successful therapeutic application of microbiome science has been the use of fecal microbiota transplantation (FMT), which involves “transplanting” stool from a healthy individual to a diseased patient, with the idea that the “healthy” microbiota will correct whatever derangement may exist in the ill patient and therefore will alleviate symptoms. Fundamentally, this notion is agnostic as to the specific microbial dysbiosis and holds that any healthy microbiota will be curative. Given its success in treating medically recalcitrant Clostridioides difficile infection (CDI) (2), FMT is now being tested in roughly 200 clinical trials (listed at ClinicalTrials.gov) for a broad range of disease indications that include CDI, inflammatory bowel disease (IBD; ulcerative colitis and Crohn’s disease), obesity, eradication of multidrug-resistant organisms, and psychiatric conditions (e.g., anxiety and depression) among others. The few published clinical studies regarding indications other than CDI have generally included small sample sizes and have offered mixed results, but these preliminary findings suggest FMT will not be effective for all diseases. In contrast to the successes in CDI, the results have been more varied for patients with IBD (3), which is perhaps the second-best-studied indication. It is not clear whether these discrepancies are due to heterogeneity in recipients (e.g., in terms of underlying disease mechanisms or endogenous microbiotas), the donor material, and/or the logistical details of FMT administration (e.g., route, frequency, and dose).

Although FMT offers an important proof of concept that microbiome-based therapies can be effective, treatment is difficult to standardize across large populations because of variability among stool donors and among the endogenous microbiotas of recipients. In addition, FMT is fraught with safety concerns, its mechanism(s) of action is unclear, and its regulatory path forward is largely uncharted. Ultimately, FMT likely represents the prototype of microbiome-based therapies; subsequent generations will include the use of more refined bacterial cocktails, single strains of bacteria, and, ultimately, bacterial factors and/or metabolites as the therapeutic intervention. The field is at the very beginning of this transition phase: several companies have bacterial cocktails, single microbes, and/or prebiotics that are currently in clinical trials (4), and the next few years will undoubtedly witness the entry of microbiome-based therapeutics into the clinic.

However, significant hurdles remain in identifying and developing future next-generation microbiome-based therapeutics. Identifying specific microbes causally related to disease protection has largely been serendipitous without a systematic approach for doing so. The challenges associated with finding this proverbial “needle(s) in a haystack” have led to the notion that communities of organisms—not specific microbes—matter most in determining disease susceptibility. This idea is rooted in the fact that the microbiome contains many emergent properties and may not represent just the sum of its constituent parts (5). However, the slowly growing number of single microbes that are effective against disease suggests there may be many cases in which a single organism may suffice as a therapeutic (6–9).

My group’s research innovatively integrates gnotobiotic murine models, microbiology, and immunology with the ultimate aim of identifying commensal bacteria that protect against inflammatory and infectious diseases. As part of this research effort, we have worked to make easier the process of identifying causal microbes. In thinking about how to improve microbiome analyses for increased specificity, we noted that microbiome-wide association studies (MWAS) are conceptually modeled after genome-wide association studies (GWAS). The issue confronted in filtering lengthy lists of MWAS results is analogous to the problem with GWAS: the identification of a long list of genes—often with no clear biological rationale—implicated in disease pathogenesis (10). GWAS began as an adjunct to classic genetic approaches to better understanding the genetic basis of complex traits; however, classic linkage studies using family pedigrees have been successful in characterizing many disease-modulating genes. Indeed, there is emerging work using family members as controls to tease out microbiome-disease relationships (11, 12). We hypothesized that adapting a pedigree analysis to study the microbiome might illuminate a pathway for pinpointing microbes that are more likely to be causally related to disease. In murine models, this is easily accomplished by cohousing mice with different microbiotas to generate “progeny” that have hybrid microbiotas reflective of both “parents.” In recent work, we demonstrated that microbe-phenotype triangulation using this type of “microbial pedigree” analysis greatly narrowed the search space of microbes associated with specific phenotypes down to a tractable number for follow-up studies (8). This bioinformatically narrowed list of taxa that are associated with a phenotype of interest facilitates add-back experiments to demonstrate causality, a feature that is challenging to do with more typical MWAS approaches that often result in >100 taxa with no clear rationale for prioritizing one over the other for mechanistic studies. Moreover, microbe-phenotype triangulation provides phenotype-directed results: unique results were obtained when the same data set was analyzed for two disparate endpoints, and the causal bacteria were only relevant to the phenotype for which they were identified (8). Although we have used this approach in the context of cohousing mice, microbe-phenotype triangulation can also be applied to human samples. Longitudinal patient samples and household controls, which are more closely related to the patient than unrelated individuals (13, 14), would provide the sort of “microbial pedigree” necessary for the analysis.

Identification of causal microbes, particularly single organisms, will open the door to the next frontier in microbiome science: characterizing the mechanism of action from the perspective of both the host and the bacterium. Elucidating the relevant host pathways and cell types required for disease protection will be critical so that the patient population can be appropriately stratified and targeted in future clinical trials. Having a single organism with a clearly defined phenotype will enable identification and characterization of bioactive microbial factors. These advances will not only increase our understanding of host-microbe interactions, but they will also serve to improve our knowledge of the underlying biology of disease pathogenesis. Given that it is not clear how generalizable the findings from these initial proof-of-concept bacteria will be, we and others in the field are beginning to employ a more global, systems-based approach to define a set of guidelines that govern productive host-microbe interactions.

**FIG 1:**
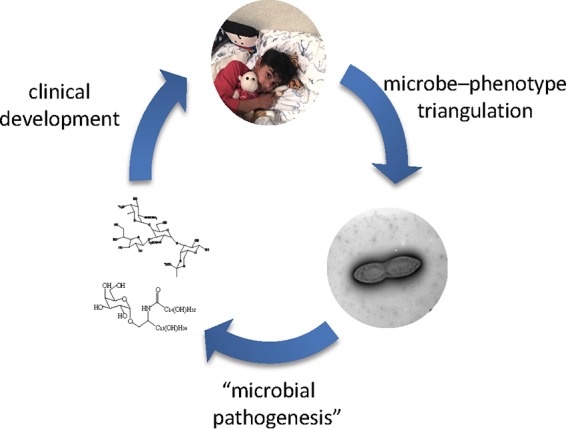
Coupling microbe-phenotype triangulation with lessons from microbial pathogenesis will yield clinically translatable discoveries (photo copyright, Neeraj K. Surana; reproduced with permission).

My group is approaching these questions by first identifying clinical issues that have significant unmet need and also have suitable animal models. We are applying the principles of microbe-phenotype triangulation to carefully selected clinical samples, isolating the identified taxa using directed culture techniques, and validating a causal microbe-phenotype relationship in animal models (Fig. 1). Using techniques that have been honed over the past century to study microbial pathogenesis, we will identify the specific bacterial molecules that modulate disease, while simultaneously elucidating the host-associated changes that are critical for their effect. Ultimately, these molecules can enter a classical drug development pipeline to develop advanced microbiome-based therapeutics.

The medical view of microbes has changed radically, moving from the early-20th-century notion that we are engaged in a constant struggle with microbes—an “us-versus-them” mentality that focused on the necessity of eradicating bacteria—to the more recent understanding that we live in a carefully negotiated state of détente with our commensal organisms. Instead of holding a simple view of microbes as enemies to be eliminated with antibiotics, scientists have increasingly recognized the critical role these organisms play in maintaining human health; loss of these host-microbe interactions in the increasingly sterile environment typical of Western civilization may have predisposed humans to the increased incidence of autoimmune and inflammatory diseases (15). As a practicing infectious disease clinician whose clinical role is to help oversee the judicious use of antibiotics, I find it ironic that we must now attempt to replicate the effects of microbes that we have spent a century trying to eliminate from the human body.

Despite initial hyperbolic hype and a few false starts, microbiome research now stands at the precipice of an ability to treat the fundamental basis of many diseases. As the field continues to mature, it will need to move beyond correlations and address causation. The identification of causal microbes and their mechanisms of action will create a “microbial toolbox” from which relevant bioactive strains can be chosen on a per-patient basis to correct specific underlying microbial dysbioses. In the near future, our knowledge base regarding the microbiome and its relationship to health and disease will be robust enough that this information can be applied in making important treatment decisions.
